# Dipodal Tetraamide Derivatives of 1,10-Diaza-18-Crown-6 and Alkylmalonic Acids—Synthesis and Use as Ionophores in Ion Selective Membrane Electrodes

**DOI:** 10.3390/s21154984

**Published:** 2021-07-22

**Authors:** Radosław Pomećko, Elżbieta Luboch, Maciej Jeszke

**Affiliations:** Department of Chemistry and Technology of Functional Materials, Faculty of Chemistry, Gdańsk University of Technology, Narutowicza Street 11/12, 80-233 Gdańsk, Poland; elzluboc@pg.edu.pl (E.L.); maciejjeszke@wp.pl (M.J.)

**Keywords:** ion-selective electrode, magnesium ion, double-armed diazacrown ether

## Abstract

Novel dipodal derivatives of an 18-membered diaza-crown ether with two diamide chains featuring methylmalonic or butylmalonic acid residues were obtained and tested as ionophores in ion-selective plasticized membrane electrodes. The objective of the study was to identify measurement conditions which ensure the most favorable performance for magnesium ion-selective electrodes. The relationship between the molar lipophilic anion salt-to-ionophore ratio and selectivity of electrodes was examined. The best result was obtained for the conventional electrode containing Mg2 ionophore. Calculated selectivity coefficients were as follows: log*K_Mg/Ca_* = −2.77, log*K_Mg/Na_* = −3.46 and log*K_Mg.K_* = −2.24 (SSM, 1M).

## 1. Introduction

The magnesium ion is one of the four most abundant in human body fluids, and it is critical for numerous cellular and biophysical functions [1]. The precise determination of magnesium ion concentration is crucial for correct diagnosis and therapies for numerous diseases [2,3]. Among the various kinds of analytical tools, spectroscopic and fluorescence methods are typical for magnesium determination [4,5], but ionic chromatography or capillary electrophoresis methods are also successfully applied in biological fluids analysis [6,7]. The potentiometric methods applying ion-selective electrodes (ISE) are also used for magnesium determination, because they are relatively inexpensive and do not need extensive sample preparation. There are some reports on magnesium selective electrodes in the literature [8,9,10] and there are also reports on intracellular measurements [11], however most of the electrodes presented still suffer from narrow working concentration ranges. The interference of calcium ions remains the major problem for magnesium selective electrodes, but it could be partially solved by use of sensor platforms [12].

Organic compounds are extensively sought after and tested so as to identify those characterized by sufficient selectivity towards Mg^2+^, and at the same time, appropriate lipophilicity to facilitate their use as ionophores in ion-selective electrodes (ISEs) used to monitor the activity of magnesium ions as an important electrolyte, e.g., in human blood [13]. The most important issue consists of the electrode’s ability to discriminate between magnesium ions and other ions such as those of calcium, sodium, and potassium.

Since the physiological blood serum/plasma concentration of free magnesium ions is 0.45–0.8 mmol/L, the required electrode selectivity coefficients are: log*K_Mg/Na_* < −3.9; log*K_Mg/K_* < −0.9; log*K_Mg/Ca_* < −2.4 (for a maximal interference of 1% by other cations) [14].

**Figure 1:**
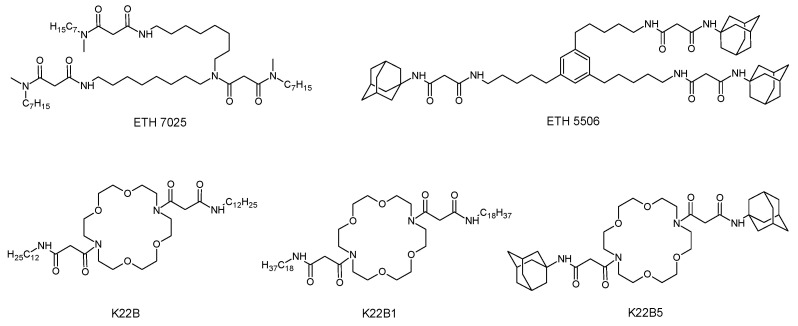
Examples of known amide magnesium ionophores of different structures [20,22,24,25].

The most important group of ionophore are the electrically neutral lipophilic oligoamide compounds, from which compounds suitable for use in ISEs selective towards magnesium ions in the presence of calcium ions are derived. Malonic acid residues are often present in these molecules. Bis(malondiamides) were the first compounds to show a noticeable preference for magnesium ions [15,16,17]. Introduction of the third arm into the molecular structure proved beneficial, as the Mg/Ca selectivity of tris(malondiamide) electrodes was shown to be ca. 10 times better that of dipodal ionophore electrodes, since the former were able to form octahedral complexes of 1:1 stoichiometry with magnesium ions [18,19,20]. An ionophore of this type is provided, e.g., by the compound referred to as ETH 7025 (Figure 1)—the commercial magnesium ionophore IV characterized by the selectivity of log*K_Mg/Ca_* = −1.2 (SSM) [21]. The addition of three secondary malondiamide units within the benzene core has significantly improved the selectivity of the electrodes towards magnesium ions as compared to calcium ions; for example, the selectivity of adamantylamide derivative referred to as ETH 5506 (Figure 1), i.e., the magnesium ionophore VI, towards Mg^2+^ ions relative to Ca^2+^, Na^+^, and K^+^ ions is log*K_Mg/Ca_* = −1.8, log*K_Mg/K_* = −2.9, log*K_Mg/Na_* = −4.1 (SSM), respectively, the ionophore’s lipophilicity amounting to log*P* = 8.48 ± 0.98 [18,22,23,24]. A new chapter in the progression of research into magnesium ionophores was opened up by Suzuki, K. et al. who described the synthesis and ionophoric properties of a several dozen compounds, including more than twenty amidic derivatives of aza-crown ethers [25]. Of these, several novel compounds were selected which, when used in ISEs, presented with selectivity towards magnesium ions. Leading selectivity properties towards magnesium were observed for the compound referred to as K22B5 (Figure 1), i.e., magnesium ionophore VII. It was suggested that the properties of K22B5 were due to interaction with the azacrown ring, which offers a lipophilic cavity of appropriate size, and diamide side chains which are capable of effectively binding the divalent cations. The additional lipophilic terminal groups of malonic amide side chanes assure the adequate surrounding to stabilize the formed complex [25]. The compound’s selectivity to Mg^2+^ ions as compared to Ca^2+^, K^+^, and Na^+^ ions was as follows: log*K_Mg/Ca_* = −2.5; log*K_Mg/K_* = −1.5; log*K_Mg/Na_* = −3.2 (SSM) while its lipophilicity was log*P* = 3.0 ± 0.4 [25]. This is the best K_Mg/Ca_ selectivity factor obtained to date.

Compared to ETH 5506, K22B5 is characterized by better selectivity towards Mg^2+^ ions as compared to Ca^2+^ ions and somewhat poorer discrimination for monovalent cations. However, to achieve such good selectivity (as with other oligoamide ionophores), K22B5 requires very precise selection of membrane composition, particularly with regard to the ratio between ionophore and salt with lipophilic anion. The most favorable molar lipophilic anion salt-to-ionophore ratio was 1:1, with the ionophore content amounting to 2% by weight of the membrane [25]. However, a disadvantage of K22B5 consists of its low lipophilicity.

Also included among the synthesized and tested 1,10-diaza-18-crown-6 derivatives were the didodecylamide (K22B) and the dioctadecylamide (K22B1) (Figure 1). These compounds present with much higher lipophilicity, albeit with poorer selectivity compared to diadamantyl derivatives (log*K_Mg/Ca_* = −1.3 and −1.2, respectively) [25].

Spichiger, E. et al. compared the properties of the K22B5 ionophore to these of its analog featuring additional methyl groups at secondary nitrogen atoms. Compound referred to as ETH 2022 was characterized by somewhat higher lipophilicity (4.44 ± 0.65 vs. 3.94 ± 0.59 for K22B5, according to the authors of [18]) but poorer selectivity coefficients (for 120% molar borate-to-ionophore ratio: log*K_Mg/Ca_* = −1.7; log*K_Mg/K_* = −0.4; log*K_Mg/Na_* = −2.4 (SSM)) [18]. Based on a theoretical model, it was assumed that K22B5 is likely to form 2:1 complexes with Mg^2+^ and Ca^2+^, while ETH 2022 tends to coordinate Mg^2+^ by forming a complex with 3:2 stoichiometry [18].

Since the K22B5, while being highly selective to magnesium ions, is poorly lipophilic, and K22B1, while being slightly less selective, is highly lipophilic, fragments of both ionophores were combined within a single molecule. The resulting ionophore was referred to as K22B1B5 and tested in magnesium-selective optodes containing the compound of interest and a lipophilic cationic dye [26].

## 2. Materials and Methods

The reagents used for the synthesis of ionophores **Mg1–Mg5** included 1,10-diaza-18-crown-6 (Synthon); BOP-Cl, diethyl methylmalonate, diethyl n-butylmalonate, adamantylamine, n-octadecylamine, n-dodecylamine, triethylamine, thionyl chloride (Sigma-Aldrich) and other typical reagents and solvents.

The reaction progress and purity of products were monitored by TLC using aluminum sheets covered with silica gel 60F_254_ (Merck, Kenilworth, NJ, USA). Reaction mixtures were separated using classical column (silica gel 60, 0.063–0.200 mm, Merck). Reagent grade solvents were used.

^1^H NMR spectra were recorded on Varian INOVA 500 spectrometer at 500 MHz. Chemical shifts are reported in δ (ppm) units. FTIR spectra (film and KBr pellet) were taken on a Nicolet iS10 apparatus. ESI mass spectra were taken on SYNAPT G2-S HDMS (Waters, Milford, MA, USA) spectrometer using a TOF mass analyzer.

RP-18 F254S 0.25 mm modified silica gel reverse phase TLC plates (20 × 20 cm) from Merck were used for the determination of ionophore lipophilicity.

Plasticizers with known lipophilicity, namely *o*-NPOE, DOS, and BBPA (Sigma-Aldrich, St. Louis, MO, USA), were used as reference.

### 2.1. Preparation of Electrodes and Potentiometric Measurements

Reagents used for electrode preparation and potentiometric measurements included tetrahydrofuran (≥99.9%), *o*-NPOE (≥99%) (Sigma-Aldrich); sodium chloride (≥99%), potassium chloride (≥99%), calcium chloride (≥98%), (POCH), magnesium chloride (≥99%) (Sigma-Aldrich), poly(vinyl chloride) (PVC, high molecular weight), potassium tetrakis(4-chlorophenyl)borate (KT*p*ClPB) (≥98%) (Fluka), poly(3,4-ethylenedioxythiophene)/poly(styrenesulfonate) blend (PEDOT/PSS, used as 1.3% (*w*/*w*) dispersion in water (conductive grade) (Sigma-Aldrich).

The aqueous solutions were prepared with deionized water of conductivity below 0.1 μS/cm, obtained using a Hydro-Lab-PL reverse osmosis (RO) station.

Potentiometric tests were carried out using a 16-channel multimeter (Lawson Labs, Malvern, PA, USA), IS 561 conventional electrode bodies (Moeller S.A., Zurich, Switzerland), glassy carbon electrodes (glassy carbon disks of 1.8 mm in diameter in poly(ether ether ketone) casing were obtained from Mineral ^®^ (Warsaw, Poland)), silver chloride reference electrode (3M KCl) with electrolyte key (1M lithium acetate) (Philips), ORION 800500U Ross Ultra D/J Reference electrode (ThermoScientific, Waltham, MA, USA, patented composition).

### 2.2. Synthesis of Novel Ionophores **Mg1**–**Mg5**

**Scheme 1:**
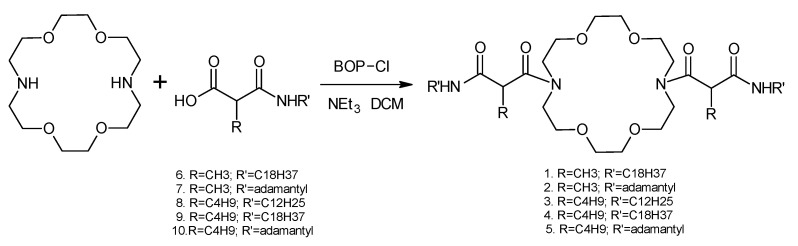
The final stage of the synthesis of ionophores **Mg1**–**Mg5** (Compounds **1**–**5**, respectively).

Synthesis of ionophores **Mg1**, **Mg2**, **Mg3**, **Mg4**, and **Mg5** (Scheme 1) was based on a literature description of preparation of similar derivatives of unsubstituted malonic acid [25], with appropriate modifications of the conditions of the various synthetic steps.

The detailed synthesis protocols and spectroscopic data could be found in the Supplementary Data. The final stage of synthesis is presented on Scheme S1.

### 2.3. Potentiometric Testing of 1,10-Diaza-18-Crown-6 Derivatives **Mg1–Mg5**—Conventional Electrodes

Cocktails were prepared containing 62.5 mg of PVC, 130 mg of *o*-NPOE, 1 wt% of ionophore and 80 to 140 mol% of KTpClPB (relative to the ionophore). The membrane components were dissolved in 1.5 mL of THF and poured onto a glass surface with a boundary defined by a 24 mm diameter ring. After evaporation of the solvent, 5 mm diameter discs were cut out from the membrane and mounted in the electrode casings. A solution of 0.1 mol/L MgCl_2_ was used as internal electrolyte. The electrodes were conditioned for 24 h in solution identical to the internal electrolyte. Prior to the measurement, the test electrodes were washed with demineralized water until the measured potential difference has stabilized.

Concentrations of the internal electrolytes and conditioning solutions were determined on the basis of the data found in [25], with the proviso that the effect of the change in concentration of the internal electrolyte in the range of 10^−4^ to 10^−1^ mol/L on the electrode parameters was tested using **Mg4** electrodes containing 1 wt% of ionophore and 100 mol% of KTpClPB relative to the ionophore. In this case, no effect of internal electrolyte concentration on electrode parameters was observed. Internal electrolyte concentration of 0.1 mol/L MgCl_2_ was maintained so that the results could be compared to the literature data. In each case, at least three electrodes of a specific membrane composition were tested. The data obtained (S, log*K_Mg/X_*) were averaged over 3 electrodes.

Selectivity coefficients were determined using the SSM method [27] with increasing concentrations of test salts. The 1 mol/L stock solutions were prepared by dissolving chloride salts of corresponding metals in deionized water and were used to prepare testing solutions by gradual dilutions. The concentrations of the testing solutions varied between 10^−6^ and 10^−1^ mol/L which led to variability ranges of activity: 10^−6^ to 6.51 × 10^−2^ and 10^−6^ to 3.82 × 10^−2^ for mono and bivalent cations, respectively when the activity coefficients were taken into account.

The following formula was used for calculating the selectivity coefficients:

(1) $$logK=\frac{E_{b}-E_{a}}{S}+\left(1-\frac{z_{a}}{z_{b}}\right)\;\log a_{a}$$

**Figure 2:**
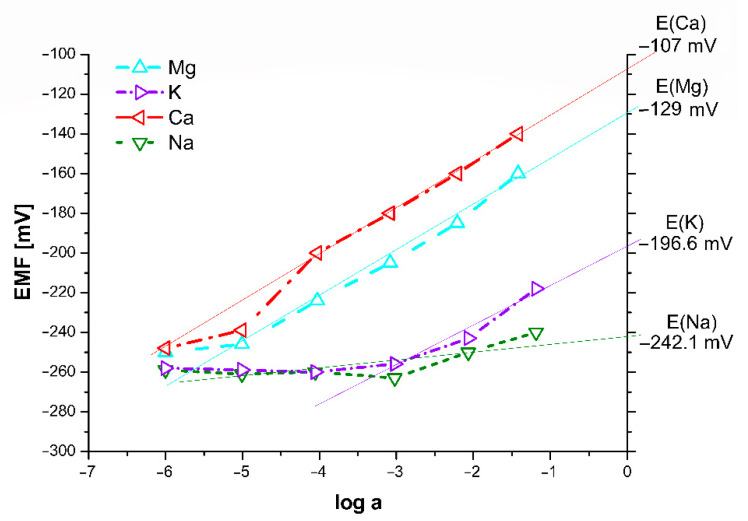
Example extrapolation of the EMF response of membrane electrode containing the ionophore **Mg4** to log(a) = 0.

Selectivity coefficients were determined for log(a) = −1 and log(a) = 0. An example extrapolation of electrode EMF response to log(a) = 0 is shown in Figure 2. Extrapolation of electrode EMF response to the primary ion activity of 1 cancels out the second part of formula 1. This method for determination of the selectivity coefficients facilitates direct comparisons of values obtained for mono- and bivalent ions, provided that all ions show typical Nernst characteristics. However, it should be stressed that Nernst slopes are not always achieved for electrode response characteristics determined for monovalent interfering ions, i.e., Na^+^, K^+^; in such cases, the course of characteristics determined for the primary ion and the interfering ion should be analyzed in addition to the selectivity coefficients being determined.

For conventional electrodes, the measurement conditions are similar to those described by Suzuki [25]. Conventional electrodes containing 1 wt% of ionophore in the membrane, as well as variable quantities of lipophilic salt, namely 80, 100, 120, and 140 mol% versus the ionophore. The selectivity coefficients for magnesium ions were determined against calcium, sodium, and potassium ions using the SSM method. Selectivity coefficients were determined for log(a) = 0 and −1. Both results were obtained by extrapolation.

**Figure 3:**
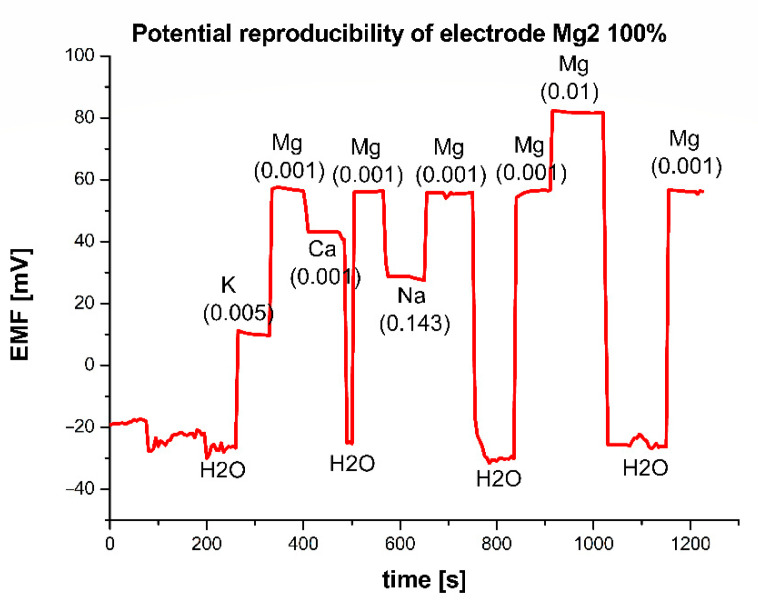
Potential reproducibility of conventional electrode in the example of electrode containing ionophore **Mg2**.

The stability of electrode’s response was tested by alternating measurement of electrode potential in MgCl_2_ solution 10^−3^ mol/L and solutions of NaCl, KCl, and CaCl_2_ at concentrations of 143.0, 5.0, and 1.1 mmol/L respectively, which are adequate to the concentrations of these ions in blood serum. The differences between potential readouts for tested 0.001 MgCl_2_ solution was not higher than 1.2 mV (Figure 3).

For the conventional electrodes characterized by the best selectivity coefficients, the additional FIM procedure was carried out. The solutions of magnesium chloride applied for the procedure varied from 10^−1^ to 10^−6^ mol/L and contained the addition of interfering Na^+^, K^+^, or Ca^2+^ ions at concentrations of 143.0, 5.0, and 1.1 mmol/L respectively.

### 2.4. Potentiometric Tests Using Glassy Carbon Solid Contact Electrodes

**Figure 4:**
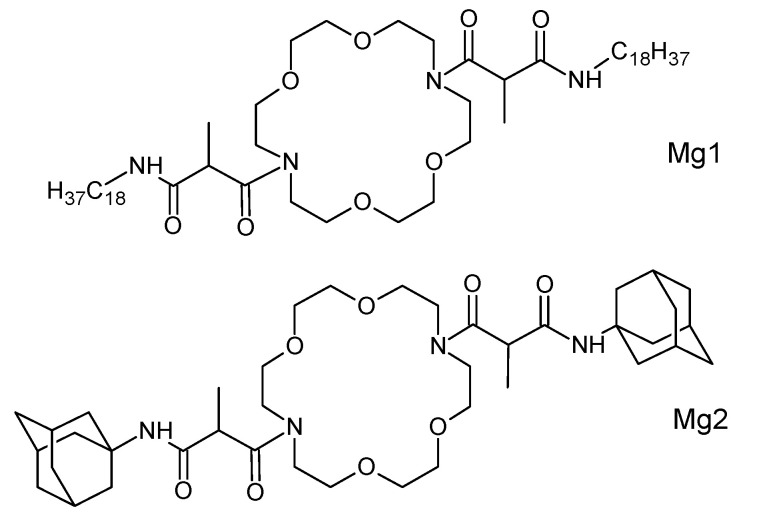

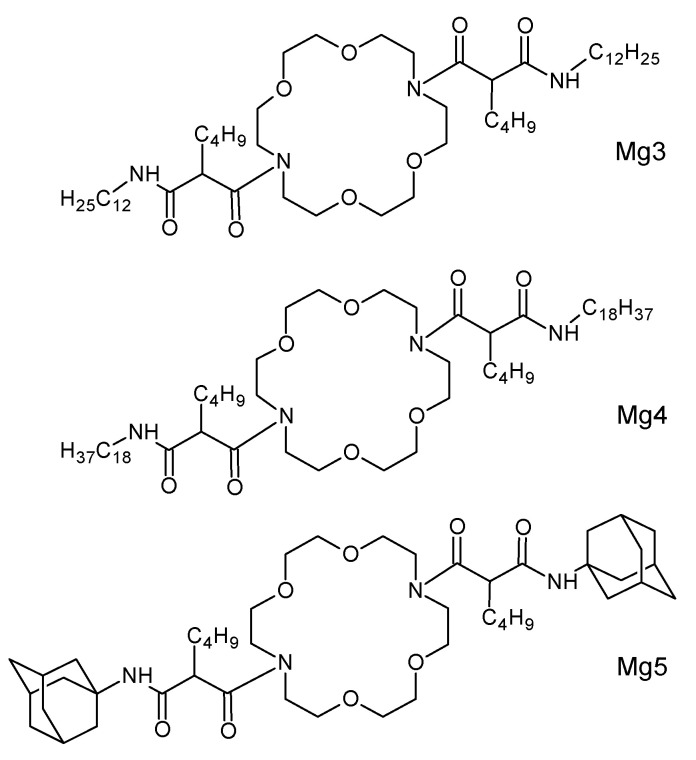
Chemical formulae for **Mg1**−**Mg5**.

Methylmalonic acid derivatives—**Mg1** and **Mg2** (Scheme 1, Figure 4) were also subjected to preliminary testing using miniature glassy carbon solid contact electrodes. Next, 20 µL of conductive PEDOT/PSS polymer was applied onto the glassy carbon surface and the electrodes were left to stay in room temperature for 24 h. Next, 20 µL of THF membrane solution (i.e., components given in Table 1 and Table 2 dissolved in 1.5 mL of THF) was applied twice from an automated pipette at the interval of 5 min. The electrodes were once again left to stay in room temperature for 24 h. For the next 24 h, the electrodes were conditioned in a 10^−3^ mol/L MgCl_2_ solution. 

For these electrodes, analyses pertained to both the percentage by weight content of the ionophore in the membrane and the molar lipophilic salt-to-ionophore ratio. Table 1 and Table 2 illustrate the composition of individual membranes containing ionophores **Mg1** and **Mg2**, respectively.

## 3. Results and Discussion

Since the popular magnesium ionophore K22B5 characterized by the best selectivity factor of log*K*_Mg/Ca_ = −2.5 [25] has low lipophilicity, its structure was modified by introduction of alkyl groups into the malonic acid residue. This type of modification of dipodal, tetraamide derivatives of 1,10-diaza-18-crown-6 has not been described to date. Compounds derived from methylmalonic and butylmalonic acid were obtained by means of synthesis in order to investigate possible direction of changes in ionophore properties displayed by this type of compound in ISEs. Synthesized analogs included not only those of K22B5, i.e., adamantyl amides, but also those of K22B1 and K22B, i.e., octadecyl and dodecyl amide analogs of ionophores described by Suzuki et al. [25]. The chemical formulae of the obtained dipodal derivatives of 1,10-diaza-18-crown-6 and their designations are given in Scheme 1 and Figure 4.

The lipophilicity of novel ionophores was determined by means of reversed phase TLC [28] using RP18 plates and 9:1 ethanol/water mixture as the chromatographic system. The estimated lipophilicity values are given in Table 3. They were compared with the values obtained for ionophores lacking the alkyl groups on α carbons within the malonic acid moieties.

**Mg1** and **Mg4** are characterized by very high lipophilicity. The lipophilicity of **Mg3** is nearly two times lower. For the remaining two compounds, lipophilicity values are below that required for ionophores to be used in ISEs for the measurement of plasma ion activity, however, marked difference in lipophilicity values exists between **Mg5** (5.3) and K22B5 (3.0).

The newly obtained amidic derivatives of 1,10-diaza-18-crown-6 and alkylmalonic acid (Figure 4) were preliminarily tested using conventional ISEs. Two of these compounds, methylmalonic acid derivatives, were also tested in miniature solid contact ISEs. Among other features, the impact of membrane composition was examined with particular reference to the molar ratio between the lipophilic anion salt and the ionophore; the impact of the wight percent content of ionophore within the membrane on the response of the electrodes in the presence of magnesium, calcium, sodium, and potassium ions was also examined. Individual examples demonstrated that the membrane’s molar lipophilic salt-to-ionophore ratio had a particular impact on the selectivity of electrodes [29,30].

Experiments involving the use of conventional ISEs were carried out to examine the properties of electrodes containing **Mg1**, **Mg2**, i.e., methylmalonic acid derivatives and **Mg3**, **Mg4**, **Mg5**, i.e., butylmalonic acid derivatives. The results of the tests are given in Table 4.

The following figures present individual selectivity coefficients for electrodes featuring **Mg1**–**Mg5** ionophores relative to the membrane’s lipophilic salt content. The values of the selectivity coefficients were calculated for log(a) = 0. The selected example responses of electrodes to variable concentrations of Mg^2+^, Ca^2+^, Na^+^, and K^+^ ions are also presented.

**Figure 5:**
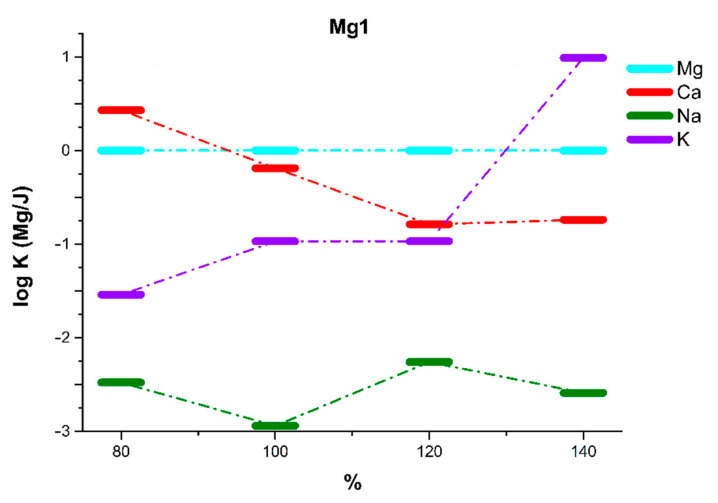
Correlation between the selectivity coefficients of conventional electrodes and the molar lipophilic salt-to-ionophore ratio for **Mg1**.

**Figure 6:**
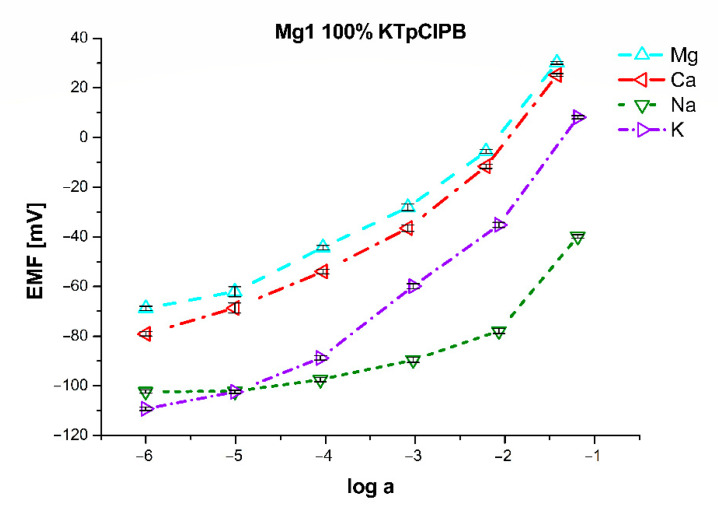
Characterization of the conventional electrode with membrane containing ionophore **Mg1** and 100% (molar) of KTpClPB.

**Figure 7:**
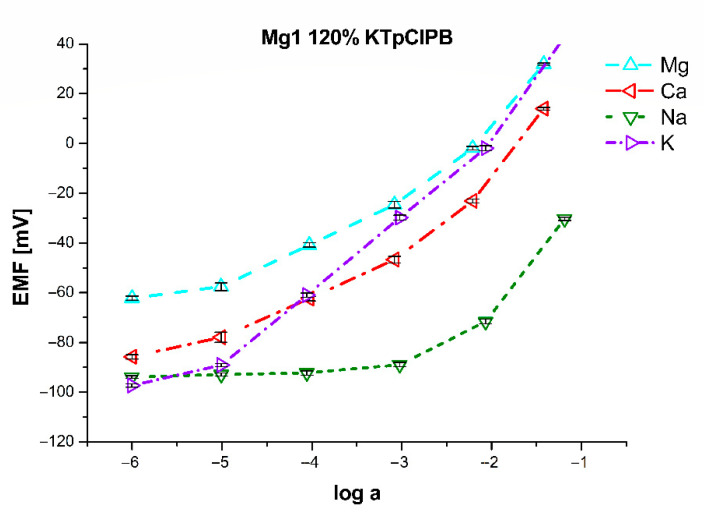
Characterization of the conventional electrode with membrane containing ionophore **Mg1** and 120% (molar) of KTpClPB.

A conventional electrode containing the highly lipophilic ionophore **Mg1** displayed a preference towards magnesium ions compared to calcium ions for lipophilic anion salt contents of 100%, 120%, and 140%. At 1 wt% membrane ionophore content and borate-to-ionophore ratio of 100 mol%, the electrode of interest can be contemplated for use in simultaneous determination of total ionized magnesium and calcium. Better selectivity towards magnesium ions compared to calcium ions was displayed by electrodes with salt-to-ionophore ratios of 120 and 140 mol%; however, strong interference from potassium ions (probably due to the cation-exchange properties of the salt itself) could already be observed at the ratio of 120% (see Figure 5, Figure 6 and Figure 7). Therefore, the electrode could be used for determination of magnesium ions only at sufficiently low potassium ion levels.

**Figure 8:**
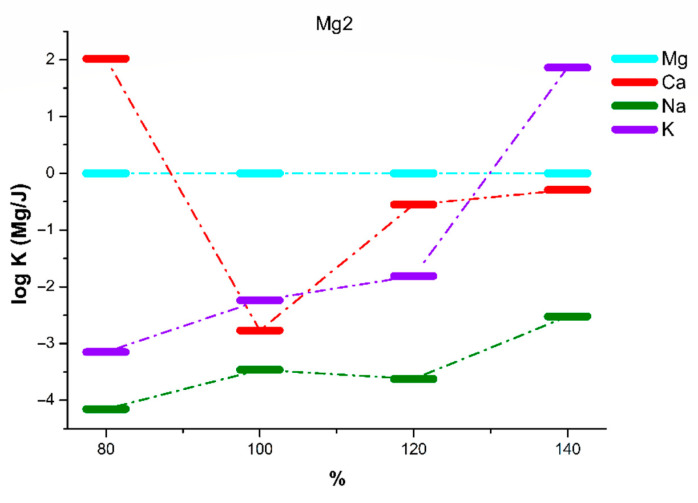
Correlation between the selectivity coefficients of conventional electrodes and the molar lipophilic salt-to-ionophore ratio for **Mg2**.

**Figure 9:**
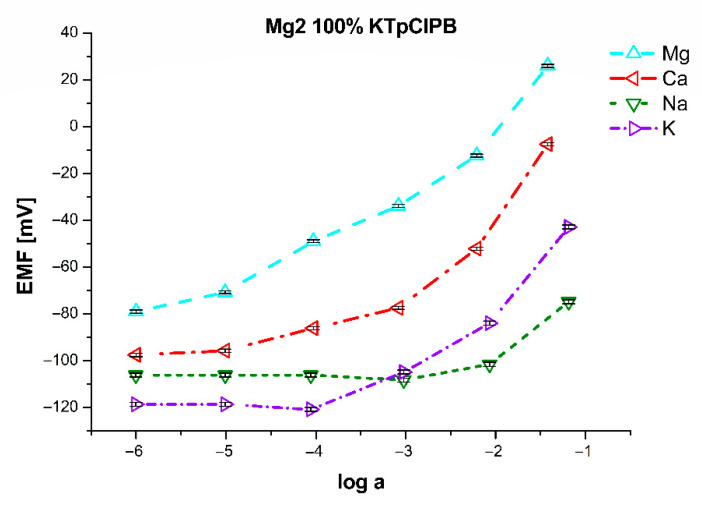
Characterization of the conventional electrode with membrane containing ionophore **Mg2** and 100% (molar) of KTpClPB.

Ionophore **Mg2** is an analog of the commercially available magnesium ionophore VII referred to in [25] as K22B5. Comparing the lipophilicity of the obtained ionophore **Mg2** to that of the parent compound K22B5 as reported in the original work, one can notice that the difference is not large (logP of 3.3 and 3.0, respectively). However, the solubility of ionophore within the lipophilic membrane is also an important criterion. **Mg2** is very well soluble in the membrane when used at quantities of up to 5 wt% (no comparison with K22B5). As in the case of K22B5, the optimum membrane composition includes equimolar amounts of lipophilic salt and the ionophore. Selectivity towards magnesium ions compared to calcium ions is similar to that of K22B5 (or slightly better depending on the determination method), and slightly better than that of sodium and potassium ions. The difference could be clearly observed at 80 mol% salt content; at this point **Mg2** started to lose its selectivity towards magnesium ions to favor calcium ions, whereas K22B5 did not do so until the salt content was as low as 60 mol%. The selectivity of **Mg2** and K22B5 towards magnesium ions was also lower at 120% salt content, but discrimination of sodium and potassium ions remained better for ionophore **Mg2** (see Figure 8 and Figure 9).

**Figure 10:**
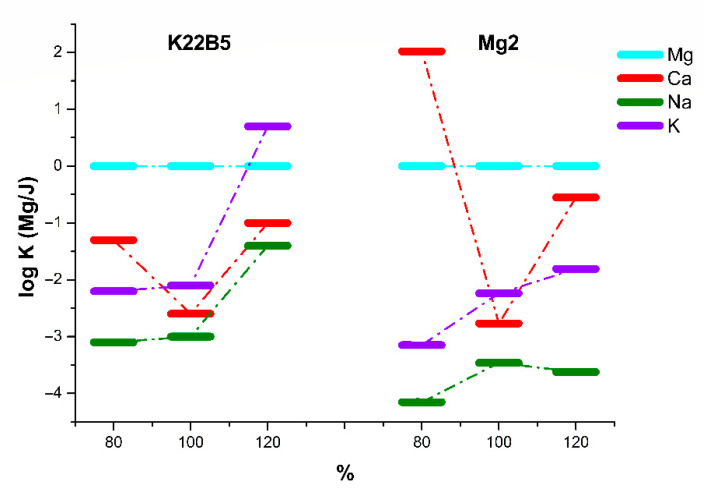
Comparison of correlation between the selectivity coefficients of electrodes and the molar lipophilic salt-to-ionophore ratios for K22B5 [18,31] and **Mg2**.

For the sake of comparison, Figure 10 also illustrates the correlation between the electrode selectivity coefficients and the molar salt-to-ionophore ratios for ionophore K22B5 [18] and for ionophore **Mg2**.

There is no doubt that the development of electrodes featuring these compounds requires a high degree of precision and that the composition of the membrane must be carefully selected to obtain the desired, favorable selectivity coefficients.

**Figure 11:**
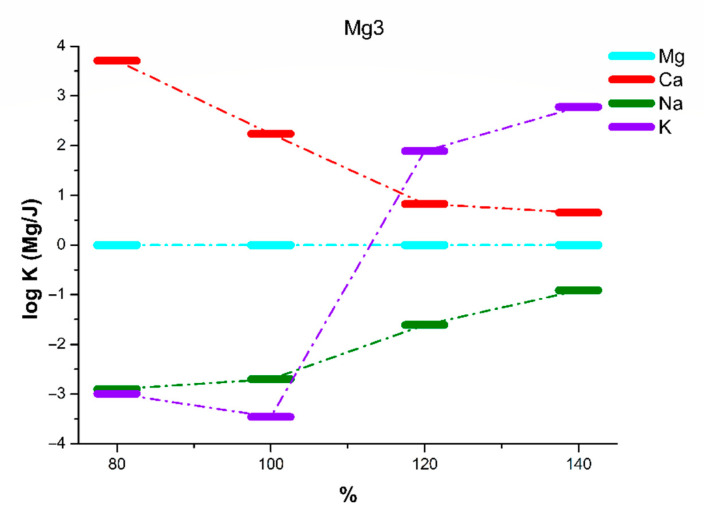
Correlation between the selectivity coefficients of conventional electrodes and the molar lipophilic salt-to-ionophore ratio for **Mg3**.

No magnesium-selective electrode was obtained to date for the **Mg3** ionophore being characterized by favorable lipophilicity (log*P* = 12). Further studies are required with this regard. For example, a calcium ionophore variant should be examined at the salt content of <80% (see Figure 11).

**Figure 12:**
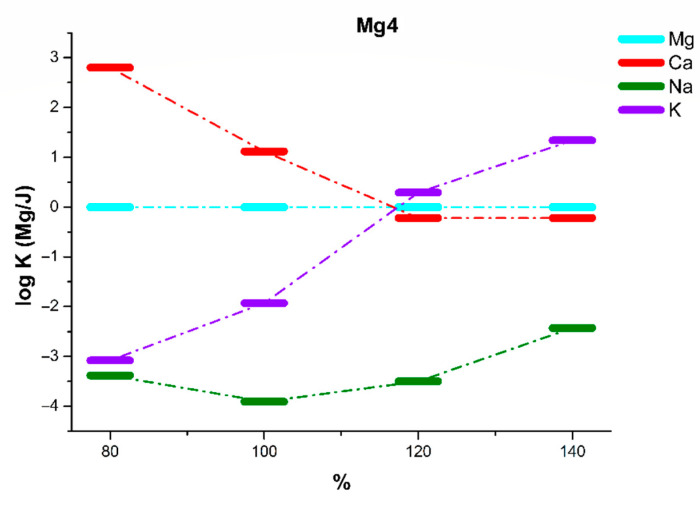
Correlation between the selectivity coefficients of conventional electrodes and the molar lipophilic salt-to-ionophore ratio for **Mg4**.

**Figure 13:**
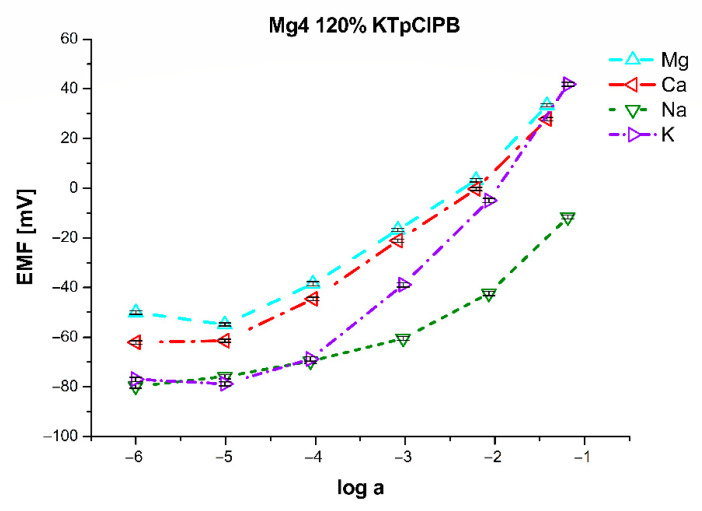
Characterization of the conventional electrode with membrane containing ionophore **Mg4** and 120% (molar) of KTpClPB.

Ionophore **Mg4**, a derivative of butylmalonic acid and n-octadecylamine with a lipophilicity of log*P* > 20, facilitates the procurement of electrodes displaying slight preference towards magnesium ions as compared to calcium ions. However, this is only the case at 120 mol% of the salt content, where no selectivity was observed in relation to potassium ions. At this salt content, the compound of interest could be a good ionophore for simultaneous determination of total magnesium and calcium ions in the absence of interference from potassium ions (see Figure 12 and Figure 13).

**Figure 14:**
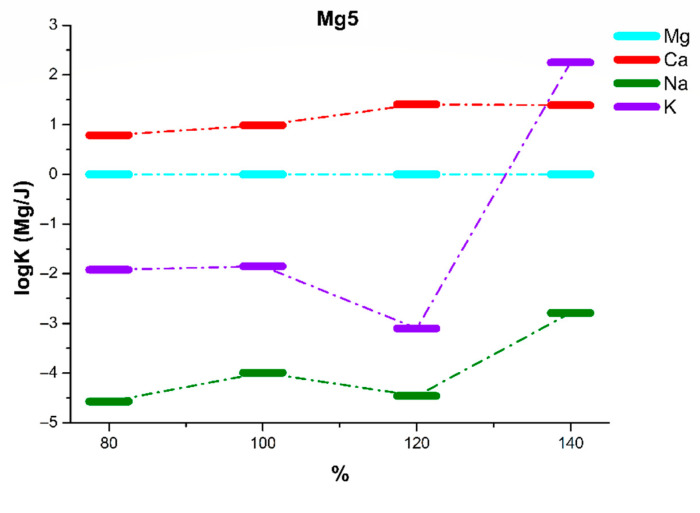
Correlation between the selectivity coefficients of conventional electrodes and the molar lipophilic salt-to-ionophore ratio for **Mg5**.

For ionophore **Mg5**, as in the case of **Mg3**, the expected results have not yet been achieved. At 120 mol% salt content, the possibility of **Mg5** being used as ionophore in electrodes dedicated to intracellular measurements could be contemplated. In the case of ionophores featuring adamantyl moieties, the cation-exchange properties of salts were observed at higher molar percentage ratios as compared to ionophores featuring long-chain amines such as n-dodecylamine or n-octadecylamine (see Figure 14).

Two of the obtained ionophores, namely **Mg1** and **Mg2**, were tested in miniature solid contact electrodes based on glassy carbon. Table 5 combines the parameters obtained for these types of electrode with membranes containing **Mg1** as the ionophore at different ionophore concentrations and variable molar salt-to-ionophore ratios. The results presented were averaged for three electrodes.

For miniature solid contact electrodes featuring the **Mg1** ionophore, the relative best performance was achieved for electrodes 1_gc_ and 4_gc_. In this case, the most favorable parameters included the membrane ionophore content of 2% and molar lipophilic salt-to-ionophore ratio not greater than 1:1 (0.8:1).

Table 6 summarizes the preliminary results of glassy carbon-based, solid contact miniature electrodes with membranes containing **Mg2** as the ionophore.

Here, the selectivity coefficient of log*K*_Mg/Ca_ = −2.62 stands out as obtained for the electrode numbered 14_sc_ (molar salt-to-ionophore ratio of 1:1, ~2% of ionophore by membrane weight). This is the optimum membrane composition for **Mg2** as tested in both conventional and solid contact electrodes, as well as for electrodes containing the K22B5 ionophore. Other selectivity coefficients are also much better than the best parameters obtained for sensors containing **Mg1**. Measurements with gc electrodes are characterized by supra-Nernst slopes of electrode performance. This is probably related to the different concentration of the conditioning solution.

**Figure 15:**
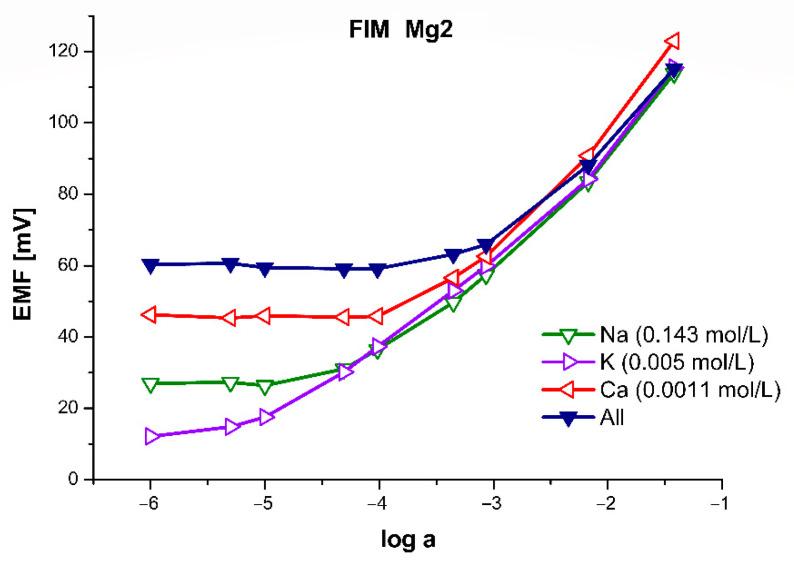
The example of FIM experiment of conventional electrode containing 1% *w*/*w* **Mg2** ionophore at 1:1 molar ratio with lipophilic salt.

The selectivity of the conventional electrodes containing 1% *w*/*w* **Mg2** ionophore with 1:1 molar ratio to lipophilic salt was also determined by FIM method (Figure 15). The electrodes proved the selectivity for Mg^2+^ cation. The calculated selectivity coefficients were as follows: log*K*_Mg/Ca_ = −1.04, log*K_Mg/Na_* = −3.76 and log*K_Mg/K_* = −2.89.

## 4. Conclusions

Several-step syntheses starting from commercially available diesters of methylmalonic and butylmalonic acids were used to obtain adamantyl, octadecyl, and dodecyl monoamides of these acids for further reaction with commercially available 1,10-diaza-18-crown-6 yielding five novel, dipodal tetraamide derivatives of this crown ether. The compounds obtained are modifications of previously described magnesium ionophores referred to by the authors of the relevant publication [25] as B22K5, B22K1, and B22K. The ionophore properties of the novel tetraamide derivatives of 1,10-diaza-18-crown-6 were examined in order to compare them to the properties of compounds known from the literature, and thus to assess the impact of alkyl substituents being introduced into the malonic acid residues on the ionophore properties of individual compounds used in ISE membranes, with particular focus on the assessment of the effect of the molar ratio between the lipophilic anion salt and the ionophore. The best result was obtained for the conventional electrode with ionophore **Mg2**, i.e., the least lipophilic of the new ionophores. For the molar lipophilic salt-to-ionophore ratio of 1:1 and 1 wt% content of ionophore within the membrane, the selectivity coefficients were obtained as follows: log*K_Mg/Ca_* = −2.77, log*K_Mg/Na_* = −3.46, and log*K_Mg/K_* = −2.24 (SSM, 1M).

We conclude that great precision is required for preparation of membranes featuring this type of ionophore. Further studies should increase the number of test membrane compositions to account for 5% of the differences in the lipophilic anion salt-to-ionophore ratios. The performance of electrodes with membranes featuring a more lipophilic plasticizer should also be examined (although, judging from the literature data, NPOE generally affords the best selectivity as is also the case for electrodes containing K22B5 [25]), with mixtures of plasticizers (e.g., NPOE with ETH 8045) also being taken into account. Further studies are also required with regard to the composition of the internal electrolyte of conventional electrodes and electrode conditioning parameters.

## Figures and Tables

**Table 1:** Compositions of the tested glassy carbon solid contact electrodes containing **Mg1** as the ionophore.

Membrane Number	KTpClPB/Ionophore (Molar Ratio)	% Ionophore by Weight	% Lipophilic Salt by Weight	% *o-*NPOE (104 mg) by Weight	% PVC (50 mg) by Weight
1.	0.4:1	2.09	0.43	65.83	31.65
2.	0.6:1	2.08	0.64	65.69	31.58
3.	0.8:1	1.02	0.42	66.56	32.00
4.	0.8:1	2.08	0.86	65.55	31.51
5.	1:1	2.08	1.04	65.43	31.46
6.	1:1	1.27	0.64	66.24	31.85
7.	1:1	3.97	1.98	63.51	30.53
8.	1.2:1	1.02	0.63	66.42	31.93
9.	1.2:1	2.07	1.28	65.27	31.38
10.	1.5:1	2.06	1.60	65.06	31.28

**Table 2:** Compositions of the tested glassy carbon solid contact electrodes containing **Mg2** as the ionophore.

Membrane Number	KTpClPB/Ionophore (Molar Ratio)	% Ionophore by Weight	% Lipophilic Salt by Weight	% *o-*NPOE (104 mg) by Weight	% PVC (50 mg) by Weight
11.	0.6:1	1.02	0.42	66.56	32.00
12.	0.8:1	2.07	1.13	65.37	31.43
13.	0.8:1	1.02	0.56	66.47	31.95
14.	1:1	2.07	1.38	65.20	31.35
15.	1:1	0.96	0.64	66.45	31.95
16.	1.2:1	1.02	0.83	66.28	31.87

**Table 3:** Lipophilicity values for the **Mg1**–**Mg5** compounds as determined by TLC, as well as the literature values for **K22B5**, **K22B**, and **K22B1** [25].

Ionophore	Log P_TLC_	Ionophore	Log P_TLC_ [25]
**Mg1**	>20	**K22B1**	15.0 ± 0.3
**Mg2**	3.3	**K22B5**	3.0 ± 0.4
**Mg3**	12.7	**K22B**	7.1 ±0.4
**Mg4**	>20	**K22B1**	15.0 ± 0.3
**Mg5**	5.3	**K22B5**	3.0 ± 0.4

**Table 4:** The effect of the molar lipophilic salt-to-ionophore ratio and the method for determination of selectivity coefficients on the values measured for conventional ISEs containing 1 wt% of ionophores **Mg1**–**Mg5**. LR—linearity range. LDL—lower detection limit. The best obtained selectivity coefficients are marked in bold.

Mg1	80% of KTpClPB		100% of KTpClPB		120% of KTpClPB		140% of KTpClPB	
LR (log(a))	−5 to −1		−5 to −1		−5 to −1		−5 to −1	
LDL (log(a))	−5.21		−5.20		−5.18		−5.19	
S (mV/dec)	24.0 ± 1.2		24.6 ± 1.1		24.0 ± 0.8		23.7 ± 1.2	
log(a)	0	−1	0	−1	0	−1	0	−1
log*K_Mg/Ca_*	0.43 ± 0.08	0.29 ± 0.08	**−0.19 ± 0.06**	−0.22 ± 0.06	−0.79 ± 0.05	**−0.81 ± 0.05**	**−0.74 ± 0.08**	−0.79 ± 0.08
log*K_Mg/Na_*	−2.48 ± 0.12	−1.66 ± 0.10	**−2.94 ± 0.13**	−1.92 ± 0.14	−2.26 ± 0.15	**−1.58 ± 0.09**	**−2.59 ± 0.12**	−1.70 ± 0.12
log*K_Mg/K_*	−1.54 ± 0.09	−0.76 ± 0.08	**−0.97 ± 0.07**	−0.12 ± 0.05	0.75 ± 0.05	1.35 ± 0.08	0.99 ± 0.09	1.56 ± 0.15
**Mg2**	**80% of KTpClPB**		**100% of KTpClPB**		**120% of KTpClPB**		**140% of KTpClPB**	
LR (log(a))	−5 to −1		−5 to −1		−5 to −1		−5 to −1	
LDL (log(a))	−5.27		−5.37		−5.34		−5.10	
S (mV/dec)	24.9 ± 1.3		20.3 ± 1.4		24.9 ± 1.2		26.7 ± 1.3	
log(a)	0	−1	0	−1	0	−1	0	−1
log*K_Mg/Ca_*	2.02 ± 0.05	1.18 ± 0.08	**−2.77 ± 0.08**	−2.49 ± 0.07	−0.55 ± 0.07	−0.73 ± 0.06	−0.29 ± 0.07	−0.29 ± 0.06
log*K_Mg/Na_*	−4.15 ± 0.09	−3.79 ± 0.10	**−3.46 ± 0.11**	−2.95 ± 0.13	−3.62 ± 0.11	−2.48 ± 0.12	−2.52 ± 0.11	−1.39 ± 0.12
log*K_Mg/K_*	−3.15 ± 0.11	−2.76 ± 0.09	**−2.24 ± 0.07**	−1.72 ± 0.09	−1.81 ± 0.10	−0.79 ± 0.09	1.86 ± 0.08	2.37 ± 0.10
**Mg3**	**80% of KTpClPB**		**100% of KTpClPB**		**120% of KTpClPB**		**140% of KTpClPB**	
LR (log(a))	−5 to −1		−5 to −1		−5 to −1		−5 to −1	
LD (log(a))	−5.34		−5.26		−5.34		−5.11	
S (mV/dec)	19.6 ± 1.1		22.0 ± 1.0		20.5 ± 1.5		22.1 ± 1.0	
log(a)	0	−1	0	−1	0	−1	0	−1
log*K_Mg/Ca_*	3.71 ± 0.12	3.00 ± 0.12	2.24 ± 0.08	1.65 ± 0.07	0.83 ± 0.06	0.59 ± 0.06	0.65 ± 0.05	0.59 ± 0.05
log*K_Mg/Na_*	−2.90 ± 0.10	−1.63 ± 0.11	−2.70 ± 0.08	−1.99 ± 0.09	−1.61 ± 0.12	−1.02 ± 0.08	−0.91 ± 0.06	−0.03 ± 0.06
log*K_Mg/K_*	−2.99 ± 0.15	−2.08 ± 0.11	−3.46 ± 0.12	−2.05 ± 0.07	1.89 ± 0.11	2.40 ± 0.12	2,78 ± 0,13	3.34 ± 0.15
**Mg4**	**80% of KTpClPB**		**100% of KTpClPB**		**120% of KTpClPB**		**140% of KTpClPB**	
LR (log(a))	−5 to −1		−5 to −1		−5 to −1		−5 to −1	
LDL (log(a))	−5.23		−5.31		−5.22		−5.32	
S (mV/dec)	23.8 ± 1.4		23.6 ± 1.2		22.9 ± 1.2		25.2 ± 1.4	
log(a)	0	−1	0	−1	0	−1	0	−1
log*K_Mg/Ca_*	2.80 ± 0.11	2.44 ± 0.11	1.12 ± 0.12	0.87 ± 0.10	**−0.22 ± 0.06**	−0.32 ± 0.05	−0.21 ± 0.06	−0.13 ± 0.05
log*K_Mg/Na_*	−3.38 ± 0.15	−1.92 ± 0.12	**−3.91 ± 0.12**	−2.53 ± 0.13	**−3.50 ± 0.11**	−2.07 ± 0.12	−2.43 ± 0.13	−1.18 ± 0.09
log*K_Mg/K_*	−3.08 ± 0.15	−1.93 ± 0.14	**−1.93 ± 0.10**	−1.18 ± 0.12	0.29 ± 0.06	0.97 ± 0.08	1.34 ± 0.12	1.92 ± 0.08
**Mg5**	**80% of KTpClPB**		**100% of KTpClPB**		**120% of KTpClPB**		**140% of KTpClPB**	
LR (log(a))	−5 to −1		−5 to −1		−5 to −1		−5 to −1	
LDL (log(a))	−5.31		−5.30		−5.34		−5.29	
S (mV/dec)	23.2 ± 1.1		21.6 ± 1.1		22.9 ± 1.3		21.4 ± 1.2	
log(a)	0	−1	0	−1	0	−1	0	−1
log*K_Mg/Ca_*	0.79 ± 0.06	0.84 ± 0.05	0.99 ± 0.05	0.96 ± 0.05	1.41 ± 0.05	1.37 ± 0.05	1.39 ± 0.12	1.29 ± 0.11
log*K_Mg/Na_*	−4.57 ± 0.10	−2.77 ± 0.12	**−3.99 ± 0.15**	−2.23 ± 0.11	**−4.46 ± 0.12**	−2.67 ± 0.10	−2.79 ± 0.05	−1.25 ± 0.06
log*K_Mg/K_*	−1.92 ± 0.10	−1.04 ± 0.08	**−1.85 ± 0.12**	−0.95 ± 0.10	**−3.10 ± 0.15**	−1.86 ± 0.10	2.25 ± 0.11	2.29 ± 0.10

**Table 5:** Parameters of miniature, solid contact, electrodes featuring **Mg1**-containing membranes (M#—number of membrane from Table 1); gc—glassy carbon solid contact electrode; selectivity coefficients determined by SSM (1M).

M#	1_gc_	2_gc_	3_gc_	4_gc_	5_gc_	6_gc_	7_gc_	8_gc_	9_gc_	10_gc_
salt/ionophore molar ratio	0.4:1	0.6:1	0.8:1	0.8:1	1:1	1:1	1:1	1.2:1	1,2:1	1,5:1
% ionophore by weight	2.09	2.08	1.02	2.08	2.08	1.27	3.97	1.02	2.07	2.06
DL (log(a))	−5.8	−4.2	−3.8	−4.6	−5.6	−4.8	−4.7	−4.2	−4.8	−4.7
S [mV/dec]	43.5	34.1	33.0	34.5	42.4	35.8	37.5	41.5	39.7	41.3
Log*K_Mg/Ca_*	**−1.65**	−1.27	−0.94	**−1.38**	−1.26	−0.43	−0.24	−0.77	−0.69	−0.84
Log*K_Mg/K_*	**−1.17**	−1.02	−1.03	**−1.51**	−0.52	−0.39	−0.16	−0.03	−0.16	−0.30
Log*K_Mg/Na_*	**−1.61**	−1.72	−1.57	**−2.13**	−1.32	−0.83	−0.70	−1.15	−0.98	−1.24

**Table 6:** Parameters of miniature, solid contact, electrodes featuring **Mg2**-containing membranes (M#—number of membrane from Table 2); gc—glassy carbon solid contact electrode; selectivity coefficients determined by SSM (1M).

M#	11_gc_	12_gc_	13_gc_	14_gc_	15_gc_	16_gc_
salt/ionophore molar ratio	0.6:1	0.8:1	0.8:1	1:1	1:1	1.2:1
% ionophore by weight	1.02	2.07	1.02	2.07	0.96	1.02
DL (log(a))	−4.1	−4.7	−4.8	−5.2	−4.4	−4.7
S [mV/dec]	44.5	45.7	37.9	43.1	41.2	41.3
Log*K_Mg/Ca_*	0.10	0.03	−0.08	**−2.62**	−1.96	0.49
Log*K_Mg/K_*	−2.69	−2.55	−0.70	**−3.54**	−1.55	−2.43
Log*K_Mg/Na_*	−2.22	−2.07	−1.25	**−2.65**	−1.48	−1.94

## Data Availability

Data is contained within the article or Supplementary Materials.

## Supplementary Materials

The following are available online at https://www.mdpi.com/article/10.3390/s21154984/s1, Scheme S1. The final stage of the synthesis of ionophores **Mg1**–**Mg5** (Compounds 1–5, respective-ly).
