# Supplementary material for: Dipodal Tetraamide Derivatives of 1,10-Diaza-18-Crown-6 and Alkylmalonic Acids—Synthesis and Use as Ionophores in Ion Selective Membrane Electrodes
Source: Sensors (Basel). 2021 Jul 22;21(15):4984. doi: 10.3390/s21154984 (PMC8348374; doi:10.3390/s21154984)
Supplement: Supplementary file 1 [file sensors-21-04984-s001.zip › sensors-1254294-supplementary.pdf]

sensors
MDPI

Suplementary data

Dipodal Tetraamide Derivatives of 1,10-Diaza-18-Crown-6 and Alkylmalonic Acids—Synthesis and Use as Ionophores in Ion Selective Membrane Electrodes

Radosław Pomećko *, Elżbieta Luboch and Maciej Jeszke

Department of Chemistry and Technology of Functional Materials, Faculty of Chemistry, Gdańsk University of Technology, Narutowicza Street 11/12, 80-233 Gdańsk, Poland; elzluboc@pg.edu.pl (E.L.); maciejjeszke@wp.pl (M.J.)
* Correspondence: radpomec@pg.edu.pl

Citation: Pomećko, R.; Luboch, E.; Jeszke, M. Dipodal Tetraamide Derivatives of 1,10-Diaza-18-Crown-6 and Alkylmalonic Acids—Synthesis and Use as Ionophores in Ion Selective Membrane Electrodes. *Sensors* **2021**, *21*, 4984. https://doi.org/10.3390/s21154984

Academic Editor: Spas D. Kolev

Received: 1 June 2021
Accepted: 15 July 2021
Published: 22 July 2021

Publisher's Note: MDPI stays neutral with regard to jurisdictional claims in published maps and institutional affiliations.

Copyright: © 2021 by the authors. Licensee MDPI, Basel, Switzerland. This article is an open access article distributed under the terms and conditions of the Creative Commons Attribution (CC BY) license (http://creativecommons.org/licenses/by/4.0/).

Synthesis and Spectroscopic Characterization of Novel Ionophores Mg1–Mg5

Synthesis of ionophores **Mg1**, **Mg2**, **Mg3**, **Mg4** and **Mg5** (Scheme S1) was based on a literature description of preparation of similar derivatives of unsubstituted malonic acid [25], with appropriate modifications of the conditions of the various synthetic steps. Commercially available diethyl esters of alkylmalonic acids — methylmalonic acid and butylmalonic acid — were used as substrates. The substrates were partially hydrolyzed with potassium hydroxide in anhydrous ethanol for 24 hours at room temperature. After the reaction mixture was acidified with concentrated HCl and extracted with diethyl ether, crude monoester monoacid compounds were obtained and further converted into monoester monoacyl chlorides using thionyl chloride. Reactions were carried out in anhydrous dichloromethane (24 h, rt). Condensation of acyl chlorides with amines (adamantylamine, n-dodecylamine, or n-octadecylamine), carried out in dichloromethane the presence of triethylamine (24 h, rt, as described in [25]) yielded monoamide monoesters of alkylmalonic acids. The resultant monoamide monoesters were hydrolyzed with sodium hydroxide in a mixture of ethanol-water (10:1) for 24 hours. After acidification of the reaction mixture, the monoamide monoacids were extracted with ethyl acetate. Compounds **6–10** were obtained (Scheme 1). The final stage of the synthesis is presented on scheme 1 and described in details in following synthesis protocols.

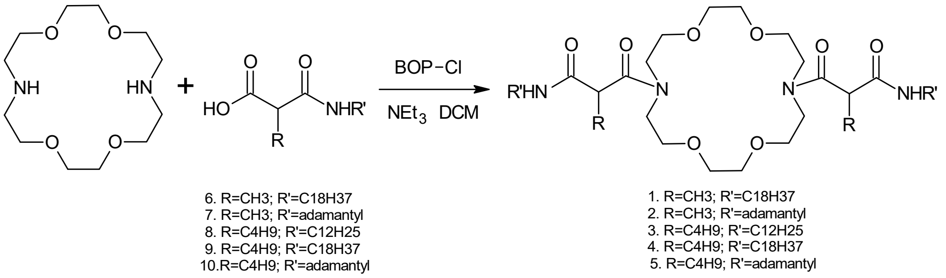

Scheme S1. The final stage of the synthesis of ionophores **Mg1–Mg5** (Compounds **1–5**, respectively).

Ionophore Mg1. 0.369 g (1.0 mmol) of compound **6** in 10 ml of DCM was cooled to 0°C. 0.24 mL (3 mmol) triethylamine, 0.266 g (1.04 mmol) BOP-Cl, and 90 mg (0.34 mmol) of 1,10-diaza-18-crown-6 were added, and the mixture was stirred at room temperature for 24 hours. Chloroform and water were added. The organic layer was separated and concentrated in vacuo and the residue was purified on a silica gel column. Ionophore **Mg1** was eluted using a 15:1 chloroform/methanol mixture. The solution was concentrated in

vacuo to yield a crystalline product which was then washed with methanol. 0.266 g (81%) of **Mg1** was obtained, mp 126–128°C.

^{1}H NMR ($CDCl_3$): 0.89 (6H, t, *J*=7.0 Hz); 1.23–1.34 (60H, m); 1.45–1.53 (10H, m); 3.13–3.21 (2H, m); 3.21–3.30 (2H, m); 3.57–3.82 (26H, m); 7.2 (2H, br). FTIR (KBr): 3273; 2919; 2851; 1648; 1560; 1468; 1437; 1368; 1260; 1215; 1115; 1077 cm^{-1}. HRMS (TOF MS ES+): [M+Na^+] 987.8054, calculated for $C_{56}H_{108}N_4O_8$+Na^+ 987.8065.

Ionophore Mg2. The synthesis was carried out in the same way as the synthesis of compound **1**, with substrate **7** being used as the starting compound. The product was eluted from the column using a 25:1 chloroform-methanol mixture. Then resultant oily substance was crystallized from methanol. 90 mg of 1,10-diaza-18-crown-6 yielded the total of 194 mg (78%) of **Mg2**, mp 154−158°C.

^{1}H NMR ($CDCl_3$) δ [ppm]: 1.41 (6H, d, *J*=7 Hz); 1.67 (12H, s); 1.97 (12H, s); 2.07 (6H, s); 3.40–3.47 (2H, m); 3.58–3.82 (24H, m); 6.72–6.76 (2H, 2m). FTIR (KBr): 3304; 2907; 2852; 1647; 1541; 1455; 1310; 1207; 1116; 1073 cm^{-1}. HRMS (TOF MS ES+): [M+Na^+] 751.4609, calculated for $C_{40}H_{64}N_4O_8$+Na^+ 751.4622.

Ionophore Mg3. The synthesis was carried out in the same way as the synthesis of compound **1**, with substrate **8** being used as the starting compound. The product was eluted from the column using a 50:1 chloroform-methanol mixture. The crystalline substance was washed with methanol and then recrystallized from DCM/MeOH as DCM was allowed to evaporate slowly. 120 mg of 1,10-diaza-18-crown-6 yielded the total of 264 mg (66%) of **Mg3**, mp 123–125°C.

^{1}H NMR ($CDCl_3$): 0.87-0.92 (12H, m); 1.24-1.36 (44H, m); 1.44-1.51 (4H, m); 1.80-1.92 (4H, m); 3.13 (2H, sextet, *J*=7.3 Hz); 3.27 (2H, sextet, *J*=7 Hz); 3.49 (2H, q, *J*=7.3 Hz); 3.56-3.82 (24H, m); 6.98-7.05 (2H, m) (2H). FTIR (KBr): 3283; 2957; 2921; 2852; 1647; 1553; 1467; 1435; 1370; 1319; 1297; 1211; 1127; 1102; 1036 cm^{-1}. HRMS (TOF MS ES+): [M+Na^+] 903.7134, calculated for $C_{50}H_{96}N_4O_8$+Na^+ 903.7126.

Ionophore Mg4. The synthesis was carried out in the same way as the synthesis of compound **1**, with substrate **9** being used as the starting compound. The product was eluted from the column with chloroform and left to crystallize from DCM/MeOH as DCM was allowed to evaporate slowly. 230 mg of 1,10-diaza-18-crown-6 yielded the total of 930 mg (100%) of **Mg4**, mp 106–110°C.

^{1}H NMR ($CDCl_3$): 0.86–0.92 (12H, m); 1.22–1.36 (68H, m); 1.43–1.51 (4H, m); 1.80–1.92 (4H, m); 3.12 (2H, sextet, *J*=7 Hz); 3.27 (2H, sextet, *J*=7 Hz); 3.49 (2H, q, *J*=7 Hz); 3.56–3.80 (24H, m); 6.98–7.05 (2H, m). FTIR (KBr): 3280; 2918; 2850; 1647; 1555; 1469; 1435; 1370; 1300; 1248; 1212; 1126; 1109 cm^{-1}. HRMS (TOF MS ES+): [M+Na^+] 1071.9020, calculated for $C_{62}H_{120}N_4O_8$+Na^+ 1071.9004

Ionophore Mg5. The synthesis was carried out in the same way as the synthesis of compound **1**, with substrate **10** being used as the starting compound. The product was eluted from the column using a 50:1 chloroform/ methanol mixture. Attempts at crystallization failed. 700 mg (98%) of compound **5** was obtained in the form of solidified oil.

^{1}H NMR ($CDCl_3$): 0.89 (6H, t, *J*=7 Hz); 2.22–1.25 (8H, m); 1.66 (12H, s); 1.78–1.86 (4H, m); 1.96 (12H, s); 2.06 (6H, s); 3.29–3.34 (2H, m); 3.56–3.68 (20H, m); 3.68–3.82 (4H, m); 6.60–6.66 (2H, m). FTIR (film): 3315; 2908; 2854; 1655; 1631; 1541; 1455; 1116; 1104; 753 cm^{-1}. HRMS (TOF MS ES+): [M+Na^+] 835.5546, calculated for $C_{46}H_{76}N_4O_8$+Na^+ 835.5561
